# Clinical and genetic features of primary ciliary dyskinesia in a cohort of consecutive clinically suspect children in western China

**DOI:** 10.1186/s12887-022-03469-x

**Published:** 2022-07-08

**Authors:** Ying Li, Wenlong Fu, Gang Geng, Jihong Dai, Zhou Fu, Daiyin Tian

**Affiliations:** 1 Department of Respiratory, Children’s Hospital of Chongqing Medical University, No. 136, Zhongshan 2nd Road, Yuzhong District, Chongqing, 400014 China; 2 Ministry of Education Key Laboratory of Child Development and Disorders, Chongqing, 400014 China; 3 China International Science and Technology Cooperation Base of Child Development and Critical Disorders, Chongqing, China; 4 Chongqing Key Laboratory of Pediatrics, Chongqing, China

**Keywords:** Primary ciliary dyskinesia, Phenotype, Gene, China

## Abstract

**Background:**

Primary ciliary dyskinesia (PCD) is a rare, inherited disorder of the motile cilia that exhibits genetic and clinical heterogeneity among different populations. PCD diagnosis remains challenging owing to the heterogeneity of associated clinical features and lack of a gold standard diagnostic test.

**Objective:**

The aim of this study was to analyze the clinical and genetic characteristics of a group of children with clinically suspected PCD in one region of China, with the goal of providing a more robust knowledge base regarding the genetic stratification underlying this disease in Chinese populations.

**Methods:**

We retrospectively analyzed the data from 38 patients with clinically suspected PCD who had undergone next-generation sequencing (NGS) between November 2016 and March 2021 in the respiratory department of a tertiary Children‘s hospital in Western China. The genetic features of the confirmed cases were summarized by reviewing data associated with other cohorts of Chinese children.

**Results:**

Overall**,** 16 patients were ultimately diagnosed with PCD with a median age of 8.5 years. All patients presented with a chronic wet cough, 93.75% exhibited chronic or recurrent sinusitis/rhinitis, 43.75% experienced recurrent wheezing, 56.25% reported respiratory symptoms present since infancy, 31.25% had a history of neonatal respiratory distress (NRD), and 25% exhibited otitis media. Only 18.75% of these patients exhibited laterality defects. High frequencies of *DNAH11* mutations were detected by integrating data from PCD patient cohorts in China.

**Conclusion:**

The high frequency of *DNAH11* mutations may limit the utility of transmission electron microscopy (TEM) as a first-line approach to diagnosing PCD in China in the absence of other indicators.

**Supplementary Information:**

The online version contains supplementary material available at 10.1186/s12887-022-03469-x.

## Introduction

Primary ciliary dyskinesia (PCD) refers to a group of related hereditary motile ciliopathies. Cases of PCD are rare, affecting just 1 per 10,000—20,000 births [1]. In childhood, abnormal motile cilia activity can result in progressive respiratory diseases that present in the form of neonatal respiratory distress (NRD), a persistent chronic wet cough often present from birth, and chronic rhinitis/sinusitis. The primary consequences of chronic airway infection and inflammation include bronchiectasis and the impairment of lung function [2]. Prior to the onset of these serious events, patients generally exhibit nonspecific respiratory manifestations that overlap with other conditions such as recurrent respiratory infections, asthma, aspiration, and immunodeficiencies. If the genetic mutations underlying PCD also result in embryonic nodal cilia dysfunction, affected individuals also exhibit a spectrum of organ laterality defects, including situs inversus totalis and situs ambiguus (SA), in which organ arrangement falls somewhere between a normal and mirrored arrangement [3, 4].

Confirmation of a PCD diagnosis remains challenging, as no single diagnostic test has been shown to offer 100% sensitivity and specificity. Historically, the diagnosis of PCD has been based on the presence of ultrastructural defects in the ciliary axoneme detected via transmission electron microscopy (TEM), but this approach is subject to significant limitations given that ~ 30% of PCD patients have normal ciliary ultrastructural characteristics [5, 6], and nonspecific ciliary changes, which can be induced by infection, may appear similar in presentation to PCD under TEM [7]. Other tests, including nasal nitric oxide (nNO) measurements, high-speed video analysis (HVSA) with ciliary beat pattern and beat frequency analyses, immunofluorescent staining of ciliary proteins, and genetic testing have emerged as alternative approaches to the diagnosis of PCD.

PCD is characterized by substantial genetic heterogeneity, with mutations present predominantly in autosomal recessive genes and less often in X-linked genes coding for axonemal, cytoplasmic, and regulatory proteins that have been implicated in this disease [6, 8]. PCD patients are thought to exhibit striking genetic stratification based on their population of origin [9–13]. In patients with mutations in specific genes associated with the pathogenesis of PCD, clinical symptoms and disease severity can vary significantly [14–16]. China is a vast country, and two pediatric PCD case series from northern and eastern China have attested to the marked genetic heterogeneity and diverse distributions among PCD patients in this country [10, 11]. The present survey of the clinical data and distributions of disease-causing mutations in a study population from Western China may thus offer valuable context for efforts to more comprehensively understand the characteristics of PCD in China.

## Methods

### Patient cohort

**Fig. 1:**
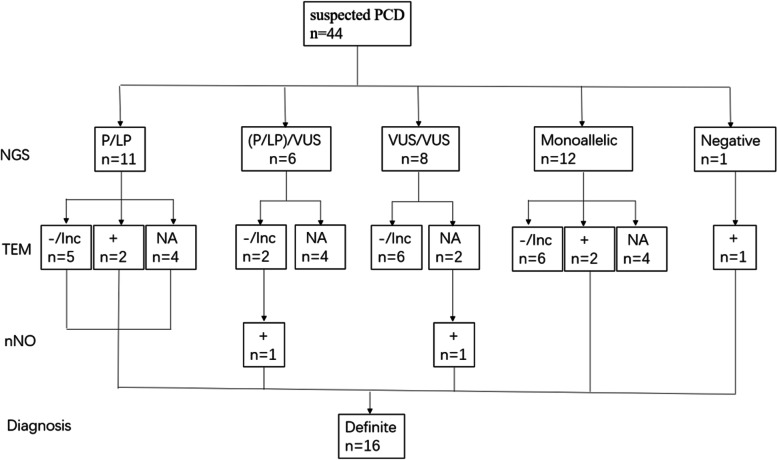
Flow chart of Reporting Trials diagram outlining the included patients. LP = likely pathogenic; nNO = nasal nitric oxide; P = pathogenic; PCD = primary ciliary dyskinesia; TEM = transmission electron microscopy; VUS = variants of uncertain significance; Inc = inconclusive; NA = notavailable; NGS = nextgenerationsequencing

In total, 44 patients with a persistent wet cough, situs anomalies, congenital cardiac defects, persistent rhinitis, chronic middle ear disease, and a history in term infants of neonatal respiratory symptoms or neonatal intensive care admission were suspected of having PCD between November 2016 and March 2021 at the Department of Respiratory Medicine, Chongqing Children’s Hospital [17, 18]. Of these patients, 38 children from 36 families underwent genetic testing, 26 underwent bronchial biopsy for subsequent TEM testing, and 2 underwent nNO measurement (Fig. 1). Demographic data pertaining to these patients including gender, age, place of residence, whether they were the offspring of a consanguineous marriage, and family history were recorded. Clinical data including whether patients presented with NRD, chronic wet cough, chronic sinusitis/rhinitis, chronic otitis media, recurrent wheezing, laterality defects, or congenital heart disease (CHD) were recorded, as was the time of the respiratory symptom onset. Additional reviewed clinical data included the age of onset for chronic wet cough, chest computed tomography (CT) results,TEM results, and genetic testing results.

### Radiological evaluation

All CT scans were performed with a Philips Brilliance iCT 128 instrument (slice 1 mm, space 1.25 mm). The internal diameter of a bronchus was measured relative to the diameter of the adjacent pulmonary artery to define bronchial dilatation, with a broncho-arterial ratio > 0.8 being defined as bronchiectasis.

### Genetic sequencing and variant assessment

A total of 16 patients underwent whole-exome sequencing, while 20 patients underwent respiratory panel analyses of more than 30 PCD-associated genes (Supplementary table 1). P4-1 and P5-1, who were the siblings of P4 and P5, respectively, underwent copy number variation sequencing (CNV-seq) and Sanger sequencing of *CCDC40* (Mygenostics Co.Ltd., China or King Medical Diagnostics Center, Guangzhou, China). Genomic DNA was extracted from peripheral blood lymphocyte samples collected from probands and their available family members. Exomes were hybridized using the GenCap probe solution. Captured DNA libraries were sequenced with an Illumina HiSeq2500 DNA Sequencer. After quality control, the clean reads were mapped to the UCSC hg19 human reference genome using Burrows-Wheelchair Aligner. All single nucleotide polymorphisms (SNPs) and indels were then identified with the GATK software (v3.4–46) or the Sentieon software (https://www.sentieon.com/), with different sequencing companies having employed different applications. Functional variant annotation was conducted using an in-house pipeline, and reported frequencies in public databases were classified into missense, nonsense, splice-site, insertion, deletion, synonymous, or noncoding mutations. Synonymous variants were discarded. The functional consequences of these mutations were predicted using SIFT, and candidate variant pathogenicity was evaluated as per the criteria of the American College of Medical Genetics and Genomics (ACMG)[19]. Variants were assessed via Sanger sequencing, with segregation analyses being conducted based upon data from available family members.

### Transmission electron microscopy

Bronchial mucosal biopsy samples were obtained through bronchoscopy. Specimens were immersed in glutaraldehyde at 4℃ for TEM (Chongqing Medical University or King Medical Diagnostics Center, Guangzhou, China). Ultrastructural defects assigned to PCD-specific ciliary defects included: 1) absent outer dynein arms alone (ODA); 2) absent outer and inner dynein arms (ODA + IDA); and 3) absent inner dynein arms with microtubular disorganization (IDA + MTD) [17, 18].

### nNO measurements

Measurements of nNO were performed in cooperative children with by inserting a nasal olive into one nostril while the other was left open. Airflow was sampled at a constant rate of 10 mL/s with velum closure using an Sunvon-CA2123. Gradual oral exhalation against resistance was performed to achieve soft palate closure recommended by ATS/ERS [20]. Three consecutive measurements were performed, and the values were averaged. For uncooperative children, nasal sampling was performed for 60 s during tidal breathing by measuring NO values for both nostrils, with the greater value being recorded. Results were reported in parts per billion (ppb).

### Statistical analysis

All statistical testing was performed using SPSS 24.0 (IBM, USA). Quantitative data were not normally distributed and were thus reported as medians with interquartile ranges. Categorical data are reported as frequencies and percentages.

## Results

### Demographic and clinical characteristics for confirmed PCD patients

In total, 16 patients from 14 families in this cohort were definitively diagnosed with PCD according to the European Respiratory Society guidelines. Of these patients, 14 exhibited biallelic or hemizygous pathogenic mutations in at least 1 PCD-related gene and/or a hallmark ultrastructural defect, while 2 patients with biallelic variants of uncertain significance in PCD-related genes and very low nNO levels were also considered to be positive for a PCD diagnosis (Fig. 1). P26 exhibited early-onset recurrent lower respiratory infections and mirror dextrocardia, but his neonatal history could not be recalled. His ciliary structure exhibited “9 + 1” microtubular organization, and genetic sequencing revealed monoallelic variants of uncertain significance in 3 PCD-related genes. This patient was highly suspected of having PCD, but we were unable to make a definitive diagnosis based on the available TEM or genetic sequencing results.

The median age of these 16 patients (62.5% male) was 8.5 years (range: 3 months—14 years). No patients were born of a reported consanguineous marriage or had a parent with diagnosed PCD. All patients were from western China (Sichuan, Yunnan, or Guizhou provinces, or Chongqing). All patients presented with a chronic wet cough, and 4 initially presented with unexplained phlegmatic sounds shortly after birth that developed into a pronounced wet cough over time. In addition, 15 (93.75%) exhibited chronic or recurrent sinusitis/rhinitis, while 7 (43.75%) experienced recurrent wheezing of whom 4 were diagnosed with asthma. Wheezing was not well controlled with standard inhaled corticosteroid (ICS) treatments in any of these patients. Additionally, 9 (56.25%) exhibited significant respiratory symptoms that developed in infancy. NRD was detected in 5 patients (31.25%), with 3 of them having been admitted to the neonatal intensive care unit for breathing support, while 2 (12.5%) could not recall whether they suffered from respiratory disease as neonates. Four patients (25%) exhibited otitis media, with 2 presenting with hearing impairments, while 3 (18.75%) exhibited situs inversus totalis (P2,P4, P5), while the siblings of P4 and P5 (P4-1 and P5-1) shared the same disease-related mutations but presented with situs solitus. One patient (6.25%) had an atrial septal defect (ASD) (Table 1), and 11 (68.75%) presented with bronchiectasis on chest CT scans, all of whom were older than 2 years of age (Table 1).

**Table 1:** Clinical features of patients

Variables	N
Total	16
Age (M, median)	102
Gender/ male	10(62.5%)
respiratory symptom onset ≤ 1 year	9 (56.25%)
chronic wet cough	16 (100%)
NRD	5(31.25%)
Recurrent wheezing	7(43.75%)
Laterality defects	3(18.75%)
Congenital heart disease	1(6.25%)
Chronic sinusitis/rhinitis	15(93.75%)
Chronic otitis media	4(25%)
**CT**	16
Bronchiectasis	11(68.75%)
Age for bronchiectasis (M, Min–Max)	79–168

*NRD* Neonatal respiratory distress, *M* Months, *Min* Minimum, *Max* Maximum

### Ciliary structural characteristics

**Fig. 2:**
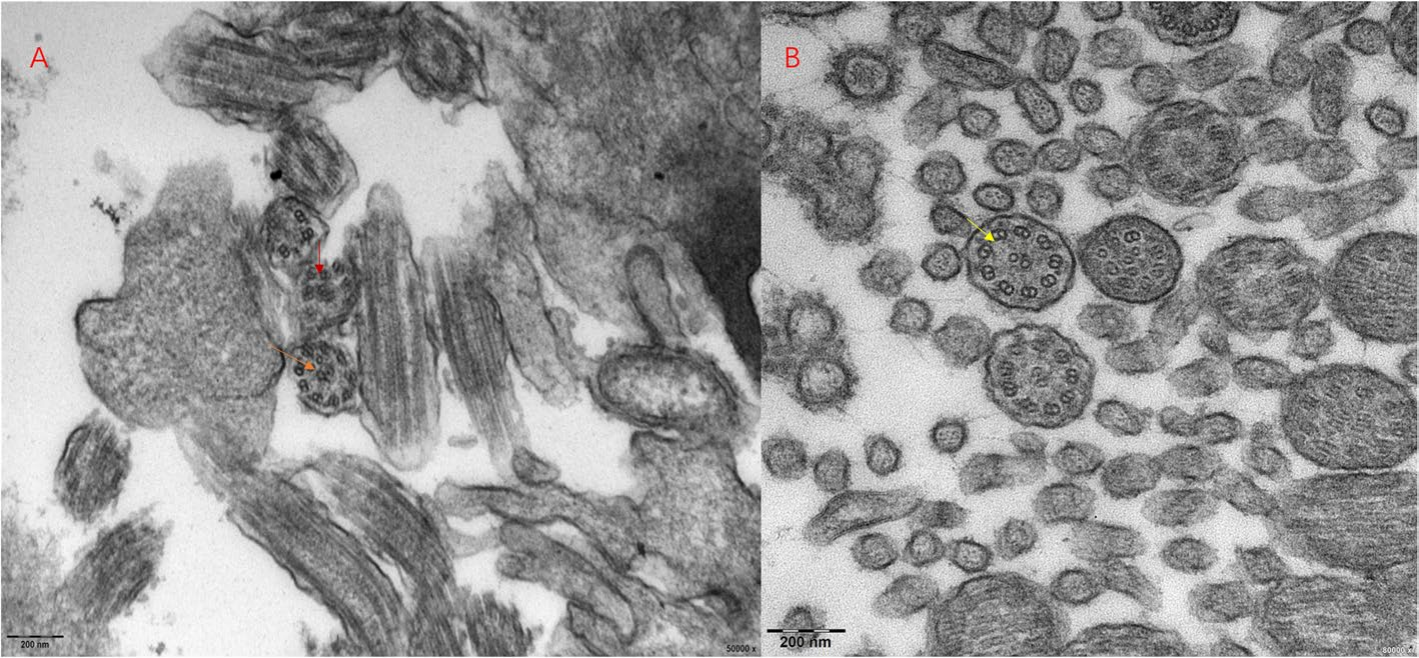
**A**-**B** Ultrastructural defects in selected patients. **A** Absence of inner dynein arm (IDA). Defect (red arrow) conjunction with central apparatus (CA) defects and microtubular disorganization(MTD) (orange arrow) in P5-1; **B** Outer dynein arm (ODA) defect and inner dynein arm (IDA) defect in P36 (yellow arrow)

Of the 26 patients that underwent bronchial biopsy, two did not undergo subsequent TEM examination as insufficient specimens were retrieved for analysis. For the remaining patients with adequate specimens, 6 exhibited normal ciliary ultrastructural characteristics of whom 3 had genetically confirmed PCD, while 4 had hallmark ultrastructural changes consistent with PCD (3 with ODA + IDA, 1 with IDA/MTD/central apparatus), and 14 had indeterminate findings (Supplementary table 2) (Fig. 2).

### Genetic characteristics

#### Genetic characteristics in the present study

In total, 38 patients underwent next-generation sequencing (whole-exome sequencing or a targeted gene panel including 30 PCD-associated genes), of whom 11 subjects from 9 families carried pathogenic/likely pathogenic (LP) biallelic or hemizygous mutations in at least one PCD-related gene, with a genetic detection rate of 28.95%. Six individuals carried pathogenic/LP variants of uncertain significance (VUS) in at least one PCD-related gene, and 8 subjects carried biallelic mutations with VUS in at least one PCD-related gene. Twelve patients exhibited heterozygous mutations in at least one PCD-related gene, of whom 2 exhibited hallmark ciliary defects and were diagnosed with PCD. One patient with hallmark ciliary defects did not exhibit any mutations in known PCD-associated genes. For the 13 PCD patients with biallelic or hemizygous genetic mutations, a total of 21 mutations spanning 10 genes (9 autosomal recessive genes and 1 X-linked gene) were identified, of which 6 (28.57%) were missense variants and 15 (71.43%) were loss-of-function mutations (nonsense, frameshift, deletion, and splice site mutations). Three subjects exhibited homozygous mutations, 2 exhibited hemizygous mutations, 8 exhibited compound heterozygous mutations. Genetic analyses revealed these subjects to harbor mutations in *DNAH11* (three subjects), *CCDC40* and *PIHID3* (two individuals each), *CCNO, DNAI2, RSPH4A, LRRC6, HYDIN*, and *RSPH9* (one individual each) (Table 2).

**Table 2:** nNO, Genetic and TEM information in This Cohort

patients	Sex	Gene	variant: coding	variant: protein	Type mutation	source	mutation on state	ACMG	nNO(ppb)	TEM
P1	F	DNAH11	c.7999C > T	p.Q2667X	Nonsense	M	Compound heterozygous t	Path	NA	Normal
		DNAH11	c.10691 + 2 T > G	NA	Splice site	P		Path		
P2	F	DNAH11	c.1295delA	p.Asn432fs	Frame shift	P	Compound heterozygous	LP	NA	NA
		DNAH11	c.1304 T > A	p.Phe435Tyr	Missense	M		VUS		
		DNAH11	c.3426-1G > A	NA	Splice site	M		Path		
P3	M	HYDIN	c.14641delG	p.V4881fs	Frame shift	M	Compound heterozygous	Path	NA	Normal
		HYDIN	c.7159-1G > A	Na	Splice site	P		Path		
		HYDIN	c.11602A > G	p.T3868A	Missense	P		VUS		
		DNAAF1	c.353-5G > A	Na	Splice site	p		VUS		
		DNAAF1	c.1300G > A	p.G434R	Missense	M		VUS		
P4	M	PIH1D3	Xq22.3(103,903,560–106,846,604) × 0	NA	Deletion	M	Hemizygous	Path	NA	MTD/CA
P4-1	M	PIH1D3	Xq22.3(103,707,506–106,837,135) × 0	NA	Deletion	M	Hemizygous	Path	NA	NA
P5	M	CCDC40	c.2677A > T	p.K893X,250	Nonsense	P	Compound heterozygous	Path	NA	NA
		CCDC40	c.993_c.994insT	p.Y332Lfs*2	Frame shift	M		LP		
P5-1	F	CCDC40	c.2677A > T	p.K893X,250	Nonsense	P	Compound heterozygous	Path	NA	IDA/MTD/CA
		CCDC40	c.993_c.994insT	p.Y332Lfs*2	Frame shift	M		LP		
P6	M	DNAI2	c.262delC	p.(Leu88fs)	Frame shift	M/P	Homozygous	LP	NA	NA
P7	F	LRRC6	c.1019dupA	p.Y340_V3 41delinsX	Nonsense	M/P	Homozygous	LP	NA	MTD
P8	M	RSPH9	c.578delC	p.P193fs*4	Frame shift	M/P	Homozygous	LP	NA	MTD/CA
P9	M	CCNO	c.267-268insAGCCC	p.V90Sfs*6	Frame shift	M	Compound heterozygous	Path	NA	Acilia
		CCNO	c.262-263insGGCCC	p.Q88Rfs*8	Frame shift	P		Path		
P10	F	RSPH4A	c.922-1G > A	NA	Splice site	M	Compound heterozygous	LP	43	Acilia
		RSPH4A	c.1069G > A	p.G357R	Missense	P		VUS		
P17	F	DNAH11	c.11498C > T	p.A3833V	Missense	P	Compound heterozygous	VUS	4	NA
		DNAH11	c.7891 T > A	p.F2631I	Missense	M		VUS		
P24	M	DRC1	c.880G > A	p.Asp294Asn	Missense	P	heterozygous	VUS	NA	ODA + IDA
		CCDC103	c.182G > A	p.Arg61Gln	Missense	P	heterozygous	VUS		
		NME8	c.416A > T	p.Asp139Val	Missense	M	heterozygous	VUS		
P25	M	HYDIN	c.7039C > T	p.(Gln2347*)	Nonsense	P	heterozygous	LP	NA	ODA + IDA
		NME8	c.1059dupA	p.(Ser354Ilefs)	Frame shift	M	heterozygous	LP		
		RSPH4A	c.2 T > C	NA	Splice site	M	heterozygous	LP		
		DNAH5	c.13286G > A	p.(Arg4429Gln)	Missense	M	heterozygous	VUS		
P36	M	Negative							NA	ODA + IDA

*IDA* Inner dynein arm, *M* Maternal, *P* Paternal, *NA* Not applicable, *ODA* Outer dynein arm, *TEM* Transmission electron microscopy, *MTD* Microtubular disorganization, *CA* Central apparatus, *Path* Pathogenic, *VUS* Variants of uncertain significance, *nNO* Nasal nitric oxide, *ppb* Parts per billion

#### Genetic characteristics of children with PCD in China

**Fig. 3:**
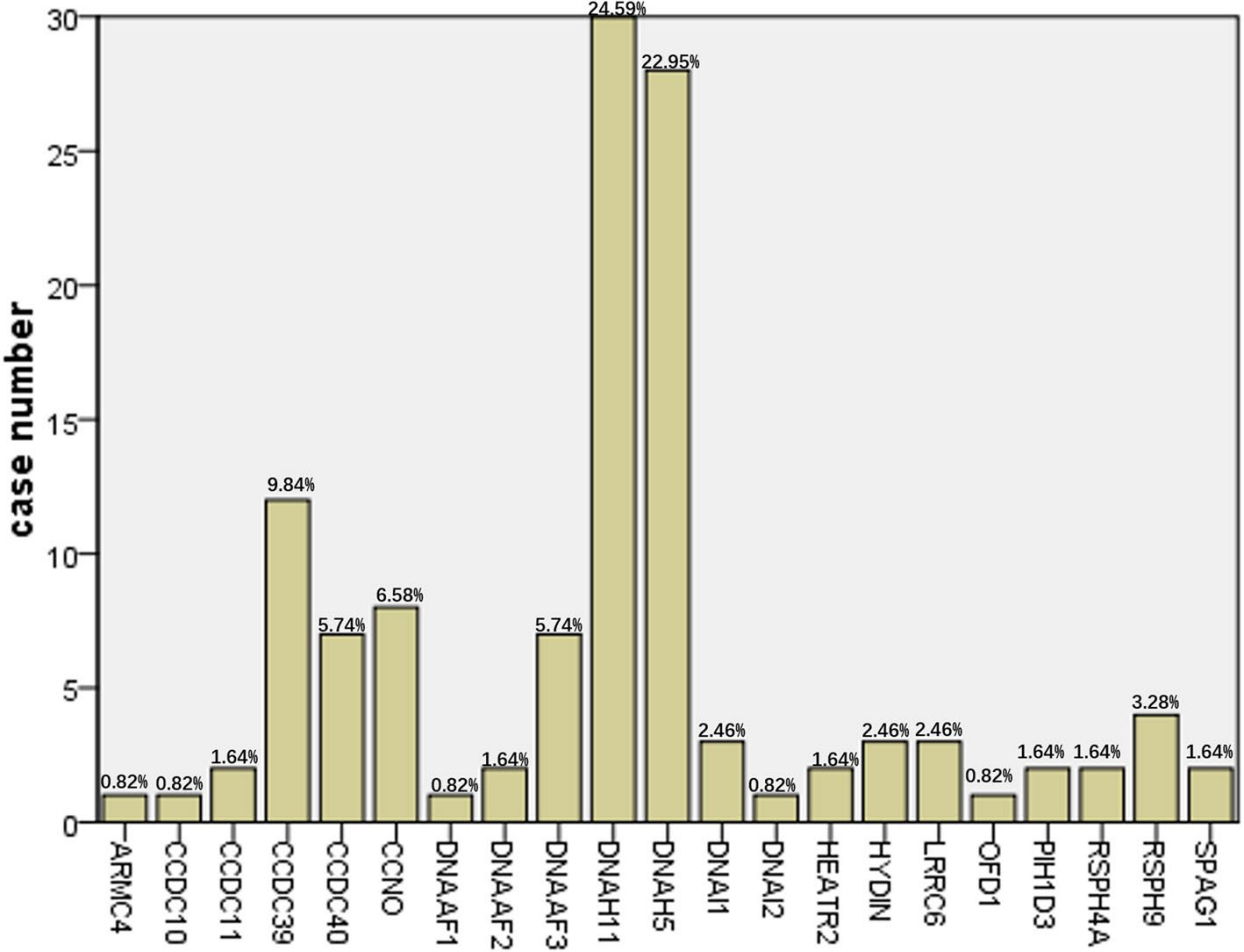
Bar graph showing the percentage distribution ofgenetic analysis in 122 individuals

The integrated genetic data from a PCD cohort of 122 Chinese patients including this cohort and those from three other studies [10, 11, 18] included 21 PCD-associated genes, and the most common pathogenic gene being *DNAH11*, followed by *DNAH5* (Fig. 3). Among the 57 identified variants in *DNAH11* in 30 patients, 59.64% (34/57) were missense.

## Discussion

In the present study, we analyzed a small pediatric PCD patient cohort in western China (Sichuan and Guizhou Provinces or Chongqing). The vast majority of patients were of Han nationality, while only two were minorities. We focused on the clinical manifestations, ciliary phenotypes, and genetic characterization of these subjects. Furthermore, we integrated the genetic results from these individuals with those from other pediatric PCD cohorts in China and gained relatively comprehensive insights into the features of the PCD genetic spectrum in China.

Clinical features of PCD including chronic wet cough, recurrent sinusitis, otitis media, neonatal respiratory distress syndrome, laterality defects, and bronchiectasis [3] were observed in this study. All of these patients had a chronic wet cough, and this high rate may stem from the bias of diseases treated in this specialty department. In our cohort, only 5% of patients presented with laterality defects, a much lower frequency than in other reports [11, 12]. A low frequency of laterality defects increases the challenges associated with the early diagnosis of PCD [21]. Laterality defects exhibit an overlapping genetic etiology with PCD and thus represent a relatively specific marker for this condition [22, 23]. However, when comparing the incidence of situs inversus (SI) (1:6000–1:8000) [24], and PCD, prior studies have concluded that most individuals affected by SI do not suffer from PCD. To date, there are 21 known PCD causative genes not associated with laterality defects [6, 25, 26]. Four individuals in the present study exhibited mutations in genes not associated with laterality defects (*HYDIN, RSPH9, CCNO, RSPH4A*). Another possible reason for the extremely low rate of laterality defects was that patients with severe organ malformation, particularly congenital heart disease, are usually referred to other specialists. Recurrent wheezing has not previously been reported as a diagnostic feature of PCD [18, 27], yet was common in the present PCD patient cohort (7/16). Prior studies have also observed high rates of recurrent wheezing or asthma in PCD patients [10]. Interestingly, ICS therapy is generally insufficient to relieve asthma symptoms in PCD patients, as shown in one study comparing airway inflammation between patients with asthma and PCD [28]. Wheezing in PCD patients likely arises due to recurrent airway infection or inflammation, although further research on this topic is warranted. Children with PCD often develop persistent respiratory symptoms that start in the first year of life [8, 27]. In previous reports, the median age of initial respiratory symptoms was 1 to 3 months [10, 13]. However, in our cohort, only 44.7% of patients reported that their initial symptoms had been present since infancy age. This may be attributable to recall bias and differences in respiratory disease severity linked to specific genes. Bronchiectasis represents a severe pulmonary sequela that can affect PCD patients, but the timing of bronchiectasis development is unclear. Prior studies have detected bronchiectasis in infants with PCD [29], while we observed it in children as young as 2 years old upon chest CT evaluation. Other reports have suggested that 70% of children with PCD present with bronchiectasis at a median age of 8 years [30]. For pediatricians, patients with chronic wet cough or recurrent wheezing should be treated cautiously, even when they do not exhibit laterality defects or bronchiectasis. Awareness of PCD among medical practitioners and taking past history into account can help avoid a delayed diagnosis [31].

Historically, TEM was a traditional test for the diagnosis of PCD, with a reported overall 86% success rate in acquiring specimens adequate for formal interpretation from nasal scrape biopsies in children [32]. In this study, this proportion was 92.3% (24/26), suggesting that mucosal biopsy via bronchoscopy was likely to be more efficient due to associated visualization and relatively integrated tissue. Three patients with definitive PCD exhibited normal ciliary ultrastructural characteristics together with mutations in *HYDIN* or *DNH11*, both of which are known to result in PCD with normal ciliary ultrastructural morphology [5, 6]. Notably, one patient found to be aciliary under TEM was ultimately determined to harbor compound heterogeneous mutations in *CCNO*, a gene related to the generation of multiple cilia [6]. As such, in the absence of genetic testing, it was sometimes hard to determine whether ultrastructural defects were primarily due to genetic mutations or were secondary to infection and/or inflammation. Moreover, improper specimen handling/processing or inexperience with the interpretation of electron microscopy results can also impact the diagnostic utility of TEM [3]. Therefore, TEM alone is not well suited to PCD diagnosis [33].

To date, more than 50 genes have been associated with PCD, and over 70% of tested patients exhibit biallelic mutations in one of these genes [8]. There is a striking genetic stratification among PCD patients with respect to their population of origin [9, 13]. Mutations in the *DNAH5* and *DNAH11* genes are thought to be the most common cause of PCD in Europeans, whereas mutations in *LRRC6* and *CCDC103* are more common in South Asian populations [9]. Genetic sequencing interpretation is not straightforward in PCD patients owing to high levels of genetic heterogeneity and allele rarity, with very little past sequencing data being available to date for Chinese patient populations that can provide important information used to exclude non-pathogenic variants. Here, we have provided clinical and genetic data for a small number of pediatric PCD patients in a single center in western China. By integrating these results with other data, we identified *DNAH11* and *DNAH5* mutations as the dominant PCD-related pathogenic variants among affected children in China. The diagnosis of patients with *DNAH11* mutations remains challenging, as mutations in this gene exhibit considerable variability in the resultant clinical phenotype [34]. In addition, PCD cases associated with *DNAH11* mutations do not exhibited any specific ultrastructural defects and thus cannot be detected via TEM [5, 35]. Therefore, the high prevalence of mutations in *DNAH11* in Chinese PCD patients may limit the utility of TEM as a first-line diagnostic approach. Moreover, locus heterogeneity and the high frequency of VUS present a substantial challenge to the translation of genomic variation to clinical practice [36], and new methods must be developed to assist in diagnosis [37, 38].

This study had a number of limitations, including a small clinical sample size and the potential for recall bias in family members when evaluating past medical history. ERS guidelines recommend nNO and HSVA testing for the initial diagnostic work-up of patients suspected of having PCD [18]. Owing to a lack of testing facilities, however, we were unable to perform any HSVA testing and nNO measurements were not taken for the majority of patients despite the importance of this screening test. In addition, some parents refused to undergo bronchial biopsy for TEM evaluation, and future studies may thus benefit from including more comprehensive diagnostic and follow-up strategies for PCD patients.

## Conclusions

In summary, we conducted an in-depth analysis of a small cohort of PCD patients from western China. Our findings were not entirely consistent with those of prior analyses, reinforcing the importance of pediatricians being made aware of PCD and paying attention to patient medical history to avoid misdiagnosing patients without other alarming features such as laterality defects. Furthermore, a high *DNAH11* variant frequency was detected by integrating the reported data from multiple regions of China, and this may y limit the utility of TEM as the first-line approach to the diagnosis of PCD.

## Supplementary Information

**Additional file 1.**

**Additional file 2.**

## Data Availability

The datasets used and/or analysed during the current study are available from the corresponding author on reasonable request.

## Abbreviations

PCD: Primary ciliary dyskinesia
NGS: Next-generation sequencing
NRD: Neonatal respiratory distress
TEM: Transmission electron microscopy
nNO: Nasal nitric oxide
HSVA: High-speed video analysis
CHD: Congenital heart disease
CT: Computed tomography
CNV-seq: Copy number variation sequencing
SNPs: Single nucleotide polymorphisms
ODA: Outer dynein arms
IDA: Inner dynein arms
MTD: Microtubular disorganization
ppb: Parts per billion
ICS: Inhaled corticosteroid
ASD: Atrial septal defect
LP: Likely pathogenic
VUS: Variants of uncertain significance
SI: Situs inversus
